# Preparation and Mechanism of Shale Inhibitor TIL-NH_2_ for Shale Gas Horizontal Wells

**DOI:** 10.3390/molecules29143403

**Published:** 2024-07-19

**Authors:** Yuexin Tian, Xiangjun Liu, Yintao Liu, Haifeng Dong, Guodong Zhang, Biao Su, Jinjun Huang

**Affiliations:** 1 Petroleum Engineering Technology Institute of Southwest Petroleum Branch, SINOPEC, Deyang 618000, China; liuyintao@sinopec.com (Y.L.); zhangguodong@sinopec.com (G.Z.); subiao@sinopec.com (B.S.); 2 State Key Laboratory of Oil and Gas Reservoir Geology and Exploitation, Southwest Petroleum University, Chengdu 610500, China; 13880093092@163.com (X.L.); donghaifeng@sinopec.com (H.D.); huangjjswpu@163.com (J.H.)

**Keywords:** shale gas, water-based drilling fluids, TIL-NH_2_, polyionic polymer, thermal stability, zeta potential, hydration inhibition, particle size distribution, electrostatic interaction, hydrogen bonding

## Abstract

In this study, a new polyionic polymer inhibitor, TIL-NH_2_, was developed to address the instability of shale gas horizontal wells caused by water-based drilling fluids. The structural characteristics and inhibition effects of TIL-NH_2_ on mud shale were comprehensively analyzed using infrared spectroscopy, NMR spectroscopy, contact angle measurements, particle size distribution, zeta potential, X-ray diffraction, thermogravimetric analysis, and scanning electron microscopy. The results demonstrated that TIL-NH_2_ significantly enhances the thermal stability of shale, with a decomposition temperature exceeding 300 °C, indicating excellent high-temperature resistance. At a concentration of 0.9%, TIL-NH_2_ increased the median particle size of shale powder from 5.2871 μm to over 320 μm, effectively inhibiting hydration expansion and dispersion. The zeta potential measurements showed a reduction in the absolute value of illite’s zeta potential from −38.2 mV to 22.1 mV at 0.6% concentration, highlighting a significant decrease in surface charge density. Infrared spectroscopy and X-ray diffraction confirmed the formation of a close adsorption layer between TIL-NH_2_ and the illite surface through electrostatic and hydrogen bonding, which reduced the weakly bound water content to 0.0951% and maintained layer spacing of 1.032 nm and 1.354 nm in dry and wet states, respectively. Thermogravimetric analysis indicated a marked reduction in heat loss, particularly in the strongly bound water content. Scanning electron microscopy revealed that shale powder treated with TIL-NH_2_ exhibited an irregular bulk shape with strong inter-particle bonding and low hydration degree. These findings suggest that TIL-NH_2_ effectively inhibits hydration swelling and dispersion of shale through the synergistic effects of cationic imidazole rings and primary amine groups, offering excellent temperature and salt resistance. This provides a technical foundation for the low-cost and efficient extraction of shale gas in horizontal wells.

## 1. Introduction

As a key formation for oil and gas exploration and development, the physicochemical properties of mud shale determine the complexity and technical difficulties of drilling engineering [1,2,3,4]. Mud shale contains a large number of clay minerals, which are prone to water-sensitive changes under the action of the aqueous phase, leading to well wall instability [5,6]. The hydration of mud shale formation is one of the main causes of well wall instability during drilling. Therefore, the study of the water sensitivity mechanism and control method of mud shale is a central factor in the field of oil and gas exploration and development [7,8]. At present, although multifunctional oil-based drilling fluids can effectively inhibit mud shale water sensitivity and have advantages such as good thermal stability and lubrication, low-cost and environmentally friendly water-based drilling fluids are gradually attracting attention because of limitations in environmental protection. Under the action of the water phase in water-based working fluids, clay minerals in mud shale will undergo a hydration reaction, causing changes in formation structure and strength [9,10]. For a long time, the influence and control of water–rock action on the mechanical properties of rock has been an important topic in the field of petroleum engineering at home and abroad, which will directly affect the safety of various oil and gas development projects in shale formations. The study of how to effectively inhibit the hydration phenomenon of shale is one of the key issues to ensure the smooth drilling of shale gas horizontal wells [11].

Under the action of water-based drilling fluids, shale water-sensitive changes are not eliminated. At present, there are more research results on the mechanism, classification, and evaluation methods of shale water-sensitive changes, so the development and design of inhibitors are the key to controlling shale water-sensitive changes [12,13]. The outstanding performance of materials such as inorganic salts [14,15], organic salts [16,17], surfactants [18,19], natural product modifications [20,21,22], and polymers [23,24,25] in inhibiting shale hydration has been confirmed by a large number of experiments, and with the continuous development of polymer drilling fluid systems, the study of polymer inhibitors has become an important topic. Among the polymer shale hydration inhibitors, researchers are increasingly focusing on polyionic liquid inhibitors [26,27,28]. This strong inhibitory water-based drilling fluid system with polyionic liquids as the core treatment agent has been widely used in domestic and foreign oilfields, and its performance is comparable to that of oil-based drilling fluids. With the deepening of related research, the structure of shale hydration inhibitors is no longer limited to a linear structure, and crosslinked polymers and hyperbranched polymers play an important role in inhibiting shale hydration swelling and dispersion because of their special spatial structure [29,30,31,32]. Therefore, the design and development of inhibitors must be closely integrated with actual engineering needs. The ability to inhibit surface hydration and also have a certain degree of salt and temperature resistance has gradually become the current trend in polymer inhibitor research [33].

The excellent performance of inhibitors is closely related to their structural characteristics. To improve the inhibition efficiency of polymer inhibitors, multifunctionality can be introduced at the synthetic design stage. In this paper, we clarify the characteristics and mechanisms of shale hydration behavior and combine this understanding with the current status and characteristics of existing polymer shale inhibitors. Our approach focuses on configuration and monomer design to enhance the structural utilization of polymer inhibitors. The inhibitor should exhibit multifunctionality to effectively inhibit the hydration behavior of ilmenite and meet the requirements for temperature and salt resistance.

We designed a new poly(ionic polymer) inhibitor based on research hotspots in the field of poly(ionic liquid) materials. This inhibitor combines inhibition and multifunctionality, enriching the variety of available inhibitors and expanding the application scope of polymers and polyionic materials in oilfield chemistry. Additionally, we analyzed the structural optimization, physicochemical properties, and action principles of the inhibitor from a chemical perspective, providing references and guidance for developing other multifunctional inhibitors with strong inhibitory properties.

## 2. Materials and Methods

### 2.1. Materials

The materials used in this study included the following: 1-Vinylimidazole, AR, Chengdu Kelong Chemical Reagent Factory; Acetonitrile, AR, Chengdu Kelong Chemical Reagent Factory; and N-(2-Bromoethyl)-1,3-propanediamine dihydrobromide, AR, Shanghai Yuanye Biotechnology Co. AR (Shanghai, China), Chengdu Kelong Chemical Reagent Factory (Chengdu, China).

### 2.2. Methods

#### 2.2.1. Infrared Spectroscopy

A 4% shale powder-based slurry was prehydrated for 24 h using a DF-101S Magnetic Stirrer (ZhuoCheng Instrument Technology Co., Ltd., Zhengzhou, China), followed by adding TIL-NH_2_ in varying ratios and blending for another 24 h. The solution was centrifuged at 8000 rpm for 5 min. The precipitate was washed with 60 mL of deionized water, dried at 105 °C for 4 h using a DF-101S Magnetic Stirrer, and ground into a fine powder. For IR analysis, 2 mg of the powder was mixed with 200 mg of KBr and pressed into pellets at 17 MPa. The spectra were recorded using a WQF-520 FTIR spectrometer (Ruili Analytical Instrument Company, Beijing, China) over a range of 400 to 4000 cm^−1^ with a resolution of 1.928 cm^−1^.

#### 2.2.2. Nuclear Magnetic Resonance Spectral Analysis

First, 12 mg of TIL-NH_2_ was dissolved in 0.5 mL of dimethylsulfoxide-d6. The n, the chemical shifts of the samples were determined by NMR spectrometry using tetramethylsilane as an internal standard. The measurements were performed with a Bruker AVANCE III HD 400 NMR spectrometer (Bruker, Fällanden, Switzerland), featuring a frequency resolution of ≤0.005 Hz and a phase resolution of ≤0.01.

#### 2.2.3. Contact Angle Analysis

First, 10 g of shale powder was dried at 105 °C for 4 h using a DF-101S Magnetic Stirrer and then pressed into cakes at 10 MPa for 5 min using a JC-TP80-5A Electronic Balance (Jingcheng Instrumentation Co., Ltd., Qingdao, China). Then, 100 mL of TIL-NH_2_ aqueous solution was prepared at different mass fractions. The shale powder cake was placed on the stage of a contact angle meter (SDC-350, Shengding Precision Instrument Co., Ltd., Dongguan, China). The solution was used to rinse the shale powder cake surface three times. A certain volume of the solution was then added to the shale powder cake using the instrument’s syringe, with the dispensing speed controlled by software. The contact angle was measured with an accuracy of ±0.1° and a resolution of ±0.01°.

#### 2.2.4. Particle Size Analysis

A 4% shale powder-based slurry was prepared and prehydrated for 24 h. Different amounts of TIL-NH_2_ were then added to the slurry and stirred for another 24 h. The particle size distribution of the suspension was tested using a BT-9300LD wet and dry laser particle size analyzer (Bettersize Instruments Ltd., Dandong, China) with a measurement accuracy of 0.6%. Before analysis, the instrument was powered on, warmed up for 1 h, and cleaned. Samples were added to the cuvette with a disposable pipette and measured. The particle size distribution curve and statistical parameters, including the median particle size (d_50_) and average particle size, were recorded.

#### 2.2.5. Zeta Potential Analysis

Prior to the assay, 12 g of shale powder was added to 300 mL of water and stirred for 24 h to prepare a basal slurry. Different concentrations of TIL-NH_2_ inhibitor (0.3–1.5%) were then added to the basal slurry and stirred for another 24 h. The slurry containing the inhibitor was tested at room temperature using a Zetaprobe Zeta Potential Analyzer (Colloidal Dynamics Inc., Ponte Vedra Beach, FL, USA) at different rotational speeds (100 to 240 rpm in 10 rpm intervals). The zeta potential was measured three times at each rotational speed, and the average value was recorded.

#### 2.2.6. XRD Analysis

First, 4 g of shale powder was added to 100 g of water and stirred for 24 h to form a suspension. Different concentrations of inhibitors were then added to the suspension, followed by stirring for another 24 h. Next, 455 mL of each suspension containing different inhibitors was added to a 505 mL centrifuge tube and centrifuged at 8000 rpm for 5 min. The precipitate was removed and washed three times with deionized water to obtain a wet shale powder sample. The X-ray diffraction spectra of the wet shale powder sample were measured using a SmartLab SE X-ray diffractometer (Rigaku Corporation, Tokyo, Japan). The X-rays used were CuKα-rays with a wavelength of 0.15406 nm, a step angle of 0.015°, a scanning range of 3° to 70°, and an angular repeatability of 5/10,000° with an accuracy of 0.005°. The wet shale powder sample was then dried at 105 °C for 24 h and ground into a dry powder. The X-ray diffraction spectra of the dry shale powder sample were measured using the same parameters.

#### 2.2.7. Thermogravimetric Analysis

First, 100 mL of TIL-NH_2_ aqueous solution at different concentrations was prepared and stirred for 24 h. Then, 4 g of shale powder was added and stirred for another 24 h for uniform dispersion. The mixture was centrifuged at 5000 rpm for 5 min, and the precipitate was dried at 40 °C for 48 h. The dried sample was ground into a fine powder. A Labsys EVO thermogravimetric analyzer (Setaram, Caluire, France) was used for analysis. The instrument was preheated for 30 min, and the temperature program was set from 40 to 800 °C at a rate of 10 °C/min. Approximately 5 mg to 10 mg of the sample was placed in a crucible, and the flow rates of the protective and purge gases were set to 20 mL/min and 50 mL/min, respectively. The test measured the sample weight change with temperature, with a weighing accuracy of 0.005% and precision of 0.0025%.

#### 2.2.8. Microstructural Analysis

A 4% shale powder base slurry (50 mL) containing different concentrations of inhibitors was centrifuged at 8000 rpm for 5 min. The precipitate was washed three times with 20 mL of deionized water. The shale powder precipitate was divided into two parts. One part was placed on a special sample stage, transferred to a vacuum chamber, rapidly frozen with liquid nitrogen at −80 °C, and dried in a vacuum freeze dryer. A conductive layer of about 20 nm was deposited on the sample surface using electron beam evaporation. The samples were observed using a Quanta 450 ambient scanning electron microscope (Thermo Fisher Scientific Inc., Waltham, MA, USA) with a magnification of 500 to 10,000 and a resolution of up to 3.0 nm in the ambient vacuum mode.

## 3. Results and Discussion

### 3.1. Synthesis of the Inhibitor TIL-NH_2_

First, 12.3 g of 1-vinylimidazole was added to 70 mL of acetonitrile solvent and then the mixture was poured into a three-necked flask equipped with a reflux condenser. The system was heated to 80 °C and 25.6 g of N-(2-bromoethyl)-1,3-propanediamine dihydrobromide was added. The reaction mixture was stirred and refluxed at 80 °C for 24 h. After the reaction, triethylamine was added to remove HBr, forming triethylamine hydrochloride. The mixture was filtered, and the precipitate was washed several times with anhydrous ethanol. The precipitate was then dried in a vacuum oven at 45 °C for 24 h, yielding a light orange solid monomer (YX-NH_2_) with a yield of 84.2%.

**Figure 1:**
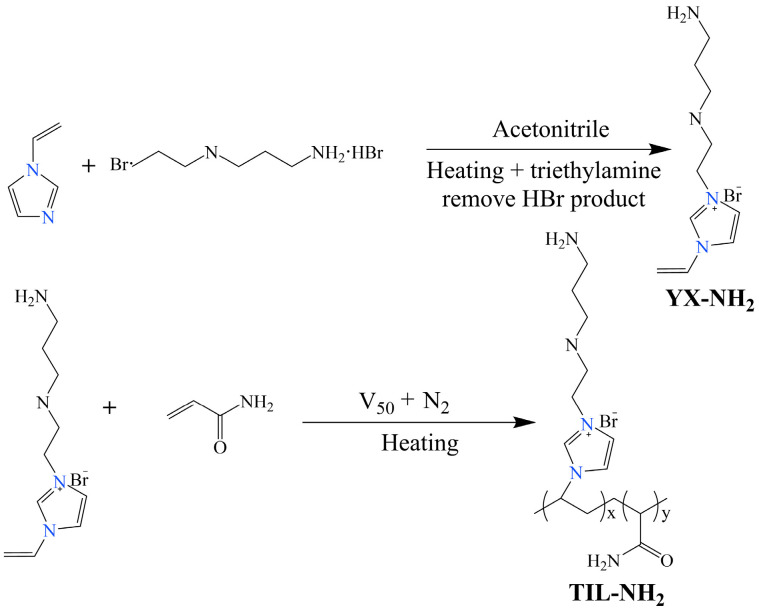
Reaction mechanism equation.

Appropriate amounts of the YX-NH_2_ monomer and acrylamide were dissolved in deionized water and placed in a three-necked flask equipped with a reflux condenser. The pH was adjusted to 5, and the mixture was heated to the desired temperature under reflux. A small amount of V-50 initiator was added under nitrogen protection, and the polymerization reaction was carried out with stirring at 200 r/min for several hours at a constant temperature. After the reaction, the mixture was transferred to a rotary evaporator and distilled under reduced pressure for 2.5 h. The resulting orange-red viscous solid product was the polyionic polymer TIL-NH_2_. The reaction mechanism is shown in Figure 1:

To assess the reproducibility of the synthesis process, we conducted three repeated experiments and recorded the yield and purity of each experiment. By calculating the yield of each experiment, we obtained the average yield and calculated its standard deviation to evaluate the differences in yield among different batches.

This section uses high-performance liquid chromatography (HPLC) to analyze the purity of each batch of TIL-NH_2_ samples. First, each batch of TIL-NH_2_ samples was dissolved in water and filtered through a 0.45 µm filter membrane to ensure that there were no particulates in the sample solution. Next, separation was performed using a C18 reverse-phase column. The mobile phase was a suitable system for TIL-NH_2_ (water–methanol system), and gradient elution was applied. The flow rate was set to 1.0 mL/min. An appropriate UV detection wavelength of 210 nm was selected to maximize the absorption signal of the target compound, and each injection volume was 10–20 µL. The samples were injected into the HPLC system, chromatograms were recorded, and the purity of the samples was calculated by comparing the peak area in the chromatogram of the sample to the peak area of the standard.

Assuming the retention time of the target product is t_R_, the peak area *A_sample_* at this time point was measured and compared to the peak area *A_standard_* of the standard. The purity was calculated using the following formula:

$$purity=\frac{A_{sample}}{A_{\mathrm{standard}}}\times 100\%$$

Each experiment was conducted following the same synthesis procedure to ensure consistency in experimental conditions. Finally, the TIL-NH_2_ from different batches was tested for its performance in inhibiting shale hydration swelling and dispersion at the same concentration. The inhibition effect of each batch was recorded and compared.

The data for yield, purity, and inhibition effect of different batches are shown in Table 1.

Based on the data in Table 1, it can be seen that the yield of TIL-NH_2_ across different batches is stable, with an average value of 84.17% and a standard deviation of 0.35%, indicating good reproducibility of the synthesis process. HPLC analysis results show that the purity of different batches is above 98%, with a standard deviation of 0.30%, indicating minimal differences in purity among batches. The inhibition effect of different batches of TIL-NH_2_ on shale hydration swelling and dispersion is consistent, ranging from 84.4% to 86.2%. These results demonstrate that the synthesis process of TIL-NH_2_ is stable and reliable and that differences in yield and purity have little impact on its performance.

### 3.2. Physicochemical Characterization of TIL-NH _2_

#### 3.2.1. Infrared Spectroscopy

**Figure 2:**
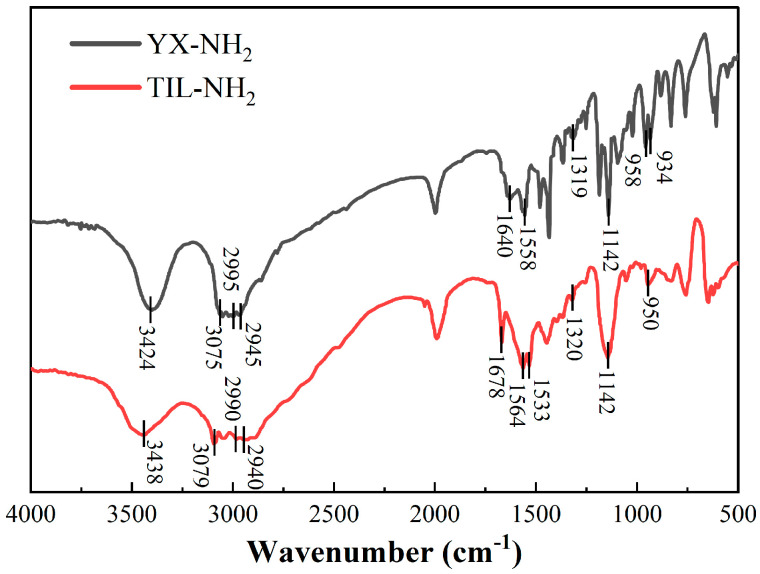
TIL-NH_2_ inhibitor infrared spectra.

The IR spectral results of TIL-NH_2_ are shown in Figure 2.

Figure 2 shows the infrared spectra of the YX-NH_2_ monomer and the TIL-NH_2_ polymer. The broad band at 3424 cm^−1^ corresponds to the stretching vibration of the N-H bond of the primary amine functional group. The peak at 3075 cm^−1^ is attributed to the stretching vibration of the C-H bond in the imidazolium ring. The peaks at 2995 cm^−1^ and 2940 cm^−1^ are due to the stretching vibrations of the C-H bonds in the methyl and methylene groups of the side chain, respectively. The peak at 1640 cm^−1^ corresponds to the stretching vibration of the C=C bond in the vinyl group. The peak at 1558 cm^−1^ is associated with the stretching vibration of the N=C bond in the cationic imidazolium ring. The peak at 1319 cm^−1^ is due to the backbone vibration of the imidazolium ring. The peak at 1142 cm^−1^ corresponds to the in-plane bending vibration of the C-H bond in the imidazolium ring. The peak at 958 cm^−1^ is attributed to the stretching vibration of the imidazolium ring, and the peak at 934 cm^−1^ is due to the in-plane swaying vibration of the C-H bond in the propylene group.

These spectral features indicate that the YX-NH_2_ monomer possesses a primary amine functional group, a cationic imidazole five-membered ring structure, and a vinyl group. Under the initiation of V-50, the vinyl group of the YX-NH_2_ monomer was attacked by free radicals, resulting in a free radical polymerization reaction that formed the TIL-NH_2_ polymer. In the infrared spectrum of the TIL-NH_2_ polymer, the spectral features of the primary amine functional group and the cationic imidazole five-membered ring remain unchanged. A new peak appears at 1678 cm^−1^, corresponding to the stretching vibration of the carbonyl C=O in the amide group (CONH2), while the spectral features of the C=C bond at 1640 cm^−1^ and the C-H bond at 934 cm^−1^ disappear. This indicates that the double bond of the vinyl group opened to form a long-chain polymer structure.

#### 3.2.2. Nuclear Magnetic Resonance Spectral Analysis

**Figure 3:**
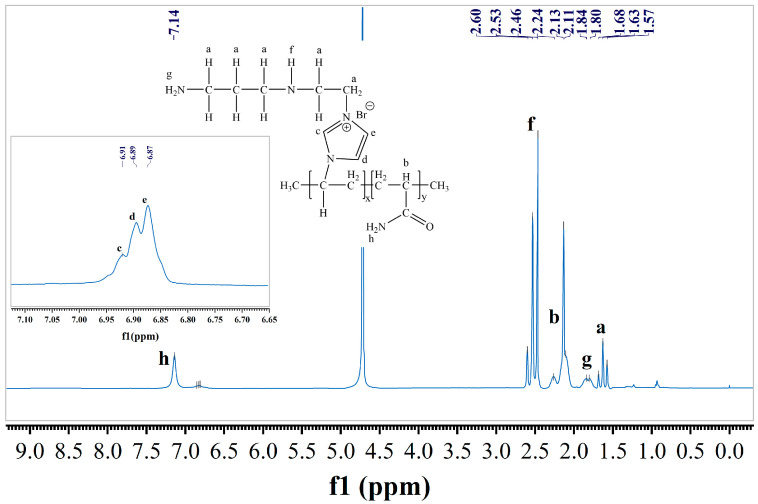
^1^H-NMR spectrum of TIL-NH_2_.

In order to characterize the structure of TIL-NH_2_ more accurately, NMR hydrogen spectroscopy was performed in this section. The measured hydrogen spectral profile is shown in Figure 3, where the letters on the chemical shift peaks correspond to the H atoms at the letters in the chemical structure formula.

As shown in Figure 3, the ^1^H-NMR spectrum of TIL-NH_2_ exhibits several distinct chemical shifts. The peaks at 1.57168 ppm (a) correspond to the hydrogen atoms of the -CH_2_- groups on the main chain of the polymer molecule. The peak at 2.24 ppm (b) is attributed to the hydrogen atoms of the -CH- groups in the acrylamide structural unit on the polymer’s main chain. The peaks at 6.87691 ppm (c, d, e) are due to the hydrogen atoms on the imidazole ring of the polymer side chain. The peaks at 2.48257 ppm (f) are attributed to the hydrogen atoms in secondary amines, while the peaks at 1.80184 ppm (g) correspond to the hydrogen atoms in primary amines. The ^1^H-NMR spectrum analysis confirms that the synthesized product is the target compound TIL-NH_2_. Compared with the broad absorption bands of primary and secondary amines at 3200 cm^−1^~3400 cm^−1^ in the infrared spectra, the ^1^H-NMR spectra provide a more accurate distinction of the primary and secondary amine functional groups in TIL-NH_2_.

#### 3.2.3. Thermal Stability Analysis

**Figure 4:**
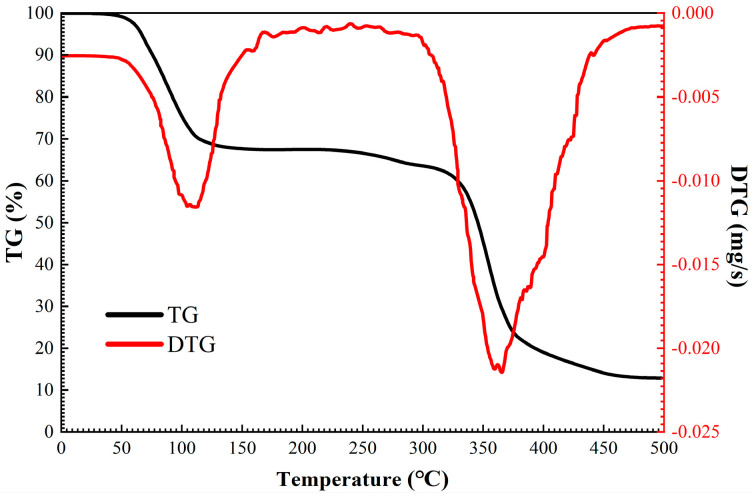
Thermal weight loss curve of TIL-NH_2_.

In order to meet the requirement of using TIL-NH_2_ as an inhibitor under high-temperature conditions in the downhole, the thermal stability test of TIL-NH_2_ was carried out in this section using a thermogravimetric analyzer. The test results are shown in Figure 4.

Based on the experimental data in Figure 4, the thermal analysis curves of TIL-NH_2_ can be divided into three temperature ranges for discussion as follows: 45 °C to 165 °C, 165 °C to 300 °C, and 300 °C to 500 °C. At around 45 °C, the sample begins to show a mass loss, with a mass loss rate of about 27.11% up to 165 °C. The mass loss rate peaks at around 100 °C, consistent with the boiling point of water. Since each unit in the molecular chain of TIL-NH_2_ contains a highly polar cationic group, it is prone to hygroscopicity, which affects the thermal analysis results.

From 165 °C to 300 °C, the TG (thermogravimetric analysis) and DTG (derivative thermogravimetric analysis) curves of TIL-NH_2_ remain basically unchanged, and the rate of mass change is lower than −0.001 mg/s, indicating that the polymer structure is stable in this temperature range. TG measures the change in mass of a material as a function of temperature, while DTG calculates the derivative of the mass change with respect to temperature, helping to identify different stages and processes of thermal decomposition.

From 300 °C to 500 °C, the molecular chain of TIL-NH_2_ starts to thermally dissociate, and the mass decreases dramatically. The rate of mass loss reaches a maximum value of about −0.026 mg/s at 365 °C. By 500 °C, the residual mass of TIL-NH_2_ is 12.76%.

### 3.3. Inhibition Mechanism Analysis of TIL-NH_2_

#### 3.3.1. Particle Size Analysis

**Figure 5:**
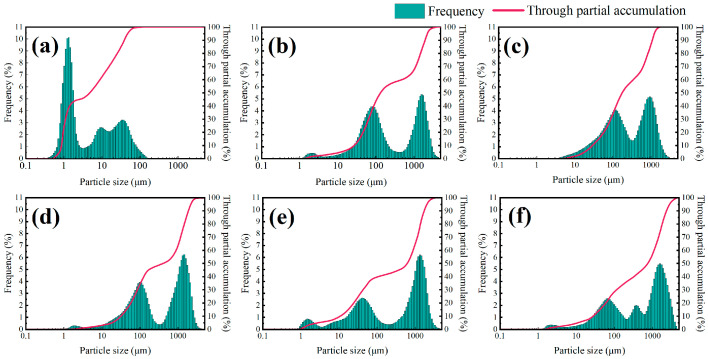
Particle size distribution of shale powder basal slurry at different TIL-NH_2_ concentrations ((**a**) 4% shale powder basal slurry, (**b**) 4% shale powder basal slurry + 0.3% TIL-NH_2_, (**c**) 4% shale powder basal slurry + 0.6% TIL-NH_2_, (**d**) 4% shale powder basal slurry + 0.9% TIL-NH_2_, (**e**) 4% shale powder basal slurry + 1.2% TIL-NH_2_, and (**f**) 4% shale powder basal slurry + 1.5% TIL-NH_2_).

**Figure 6:**
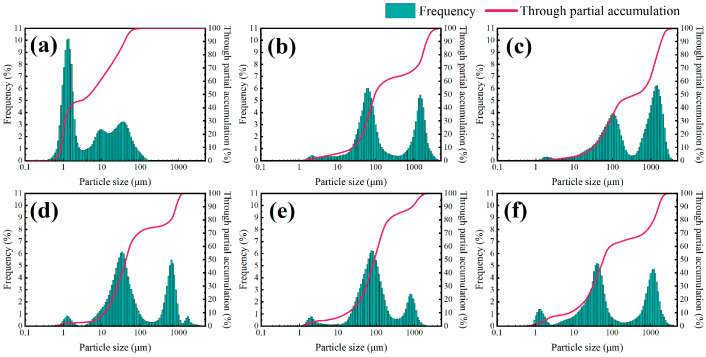
Particle size distribution of the shale powder matrix after the addition of each inhibitor ((**a**) 4% shale powder matrix, (**b**) 4% shale powder matrix + 2% DEM, (**c**) 4% shale powder matrix + 0.9% TIL-NH_2_, (**d**) 4% shale powder matrix + 5% KCl, (**e**) 4% shale powder matrix + 2% polyether amine, and (**f**) 4% shale powder matrix + 2% NW-1).

The experimental results of testing the particle size distribution in this section are shown in Figure 5 and Figure 6.

Figure 5 shows the particle size distribution curves of a 4% shale powder matrix and the addition of TIL-NH_2_ at different concentrations. Compared with the shale powder matrix, the addition of TIL-NH_2_ significantly decreased the particle content in the 0.1 μm and 10 μm ranges, shifted the particle content in the 10 μm~100 μm range to the right, and increased the particle content in the over 100 μm range, with a new peak appearing. This indicates that TIL-NH_2_ altered the dispersion state of particles in the shale powder matrix, promoting particle aggregation and inhibiting particle hydration and dispersion.

As the concentration of TIL-NH_2_ increased, the content of particles in the 10 μm~100 μm range continued to decrease, while the content of particles over 100 μm continued to increase, reflecting an increase in both the average and median particle size of the shale powder matrix. The polyionic polymer TIL-NH_2_ can aggregate surrounding shale powder particles by bridging them through the polymer molecular chains, showing higher aggregation efficiency compared with small-molecule ionic liquids. When the concentration of TIL-NH_2_ exceeded 0.9%, the average and median particle sizes of the shale powder base slurry exceeded 300 μm, effectively suppressing the hydration and dispersion of shale powder.

Additionally, the figure shows that the particle size accumulation curve of the shale powder matrix is significantly higher than that after adding TIL-NH_2_, indicating that the particle distribution in the shale powder matrix is more uniform. In contrast, the particle distribution after adding TIL-NH_2_ is more uneven, with larger particle aggregates, suggesting that TIL-NH_2_ significantly inhibits the hydration and dispersion of shale powder.

Figure 6 shows the particle size distribution curves and particle size accumulation curves of a 4% shale powder matrix with different concentrations of inhibitors. The figure demonstrates that all types of inhibitors can improve the hydration and dispersion phenomena of particles in the shale powder matrix. Among the inhibitors, 5% KCl and 2% NW-1, as small-molecule cationic inhibitors, increased the median particle size of the shale powder matrix from 5.2871 μm to 57.9824 μm and 40.7652 μm, respectively, with similar inhibition effects.

DEM and polyether amine, as amine polymers, more effectively inhibited the hydration and dispersion of particles in the shale powder matrix, with median particle sizes reaching 73.3025 μm and 63.5874 μm, respectively. The imidazole cation and amine group are two inhibitory functional groups in the linear polymer TIL-NH_2_, which can effectively inhibit the hydration and dispersion of particles in the shale powder matrix. Consequently, the median and average particle sizes for 0.9% TIL-NH_2_ were higher than those for the other inhibitors, with both values exceeding 320 μm.

Additionally, the particle size distribution curve and the particle size accumulation curve of the shale powder matrix were lower than those with the added inhibitor. This indicates that the particles in the shale powder matrix were more homogeneously distributed, while the particles became more unevenly distributed with the addition of the inhibitor, forming larger particle aggregates. This demonstrates that the inhibitor significantly inhibited the hydration and dispersion of shale powder.

#### 3.3.2. Contact Angle Analysis

**Figure 7:**
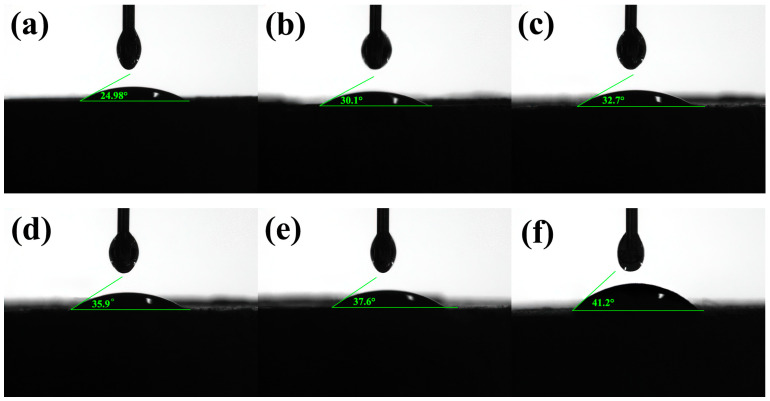
Contact angles of different concentrations of TIL-NH_2_ on a shale powder cake ((**a**) clear water, (**b**) 0.3% TIL-NH_2_, (**c**) 0.6% TIL-NH_2_, (**d**) 0.9% TIL-NH_2_, (**e**) 1.2% TIL-NH_2_, and (**f**) 1.5% TIL-NH_2_).

The contact angle of the TIL-NH_2_ aqueous solution with a shale powder cake measured in this section is shown in Figure 7.

Figure 7 shows that the contact angles of the TIL-NH_2_ inhibitor solutions are all less than 90°, indicating that water is wettable to shale. Since the main clay mineral of shale powder is illite (64.5%), the inhibitor does not affect the wetting state of water on shale. With pure water, the contact angle between the water and the shale powder cake was 24.98°. After adding TIL-NH_2_, the contact angles increased, indicating that the inhibitor attenuates the wetting and intrusion effect of water on illite.

When the concentrations of TIL-NH_2_ were 1.2% and 1.5%, the contact angles increased to 37.6° and 41.2°, respectively, which were 50.5% and 64.9% higher than pure water. This indicates that TIL-NH_2_ increases the hydrophobicity of the shale surface, forming a hydrophobic film that prevents water molecules from adsorbing and invading the illite. The hydrophobic film reduces the hydration force of illite, decreasing the wettability of water on shale.

As an inhibitor, TIL-NH_2_ reduces the wetting and intrusion rate of water on shale, prolonging the formation time of a mudcake and helping reduce filtration loss. The increase in contact angle reflects an increase in solid–liquid interfacial tension and surface energy, making it harder for water molecules to move to the shale surface, thereby improving the inhibition capability.

To evaluate the stability of the hydrophobic film on the shale surface treated with TIL-NH_2_ after long-term exposure to water and other drilling fluids, the following experiment was conducted. First, shale samples were prepared and cleaned with deionized water and ethanol to ensure the surface was free of dirt and impurities. The samples were then immersed in a 1 wt% TIL-NH_2_ solution for 24 h to form a uniform hydrophobic film. The treated samples were subsequently immersed in deionized water and various drilling fluids (water-based drilling fluid, oil-based drilling fluid, and synthetic-based drilling fluid), with temperature (25 °C) and humidity controlled in an incubator to simulate actual drilling conditions. At predetermined soaking times (1 day, 7 days, 14 days, and 30 days), the samples were removed and dried, and their contact angles were measured using a contact angle meter. Measurements were taken at multiple locations on each sample to reduce error. The experimental results are shown in Table 2.

(1) Short-Term Stability (1–7 Days):

In the short term, the contact angles of all samples showed a slight decrease, but overall changes were minimal. The contact angles in the deionized water and various drilling fluids decreased from the initial 41.2° to 40.8°–40.5° after 1 day and to 39.5°–39.2° after 7 days. This result indicates that TIL-NH_2_ maintains good hydrophobicity in the short term, with stable inhibition effects.

(2) Long-Term Stability (14–30 Days):

After longer soaking times, the contact angles decreased significantly. After 14 days, the contact angles in the deionized water decreased to 37.0°, in the water-based drilling fluid to 36.8°, in the oil-based drilling fluid to 37.2°, and in the synthetic-based drilling fluid to 36.9°. After 30 days, the contact angles further decreased to 33.5°–33.2°, indicating that the stability of the hydrophobic film gradually weakened over time.

(3) Effect of Different Drilling Fluids:

The impact of different drilling fluids on the stability of the hydrophobic film showed some differences. Overall, the contact angle changes were the smallest in the oil-based drilling fluid, indicating that the oil-based drilling fluid had the weakest erosive effect on the hydrophobic film. The water-based and synthetic-based drilling fluids had similar impacts, both leading to significant decreases in contact angles, while the effect of the deionized water was intermediate.

The reasons for the decrease in hydrophobicity include the gradual erosion of the TIL-NH_2_ hydrophobic film by water molecules over long-term soaking, which damages its integrity and hydrophobicity, and the chemical components in water-based and synthetic-based drilling fluids interacting with TIL-NH_2_, weakening the stability of the hydrophobic film. In contrast, the oil-based drilling fluid remains relatively stable as it does not contain water molecules, reducing competitive interactions with the hydrophobic film. In the short term (1–7 days), the hydrophobic film on the shale surface treated with TIL-NH_2_ effectively inhibits water penetration and shale hydration swelling, ensuring short-term inhibition effects. However, in the long term (14–30 days), as hydrophobicity decreases, the inhibition effect of TIL-NH_2_ may weaken, indicating that additional measures are needed to ensure the effectiveness of TIL-NH_2_ during extended drilling operations.

#### 3.3.3. Zeta Potential Analysis

**Figure 8:**
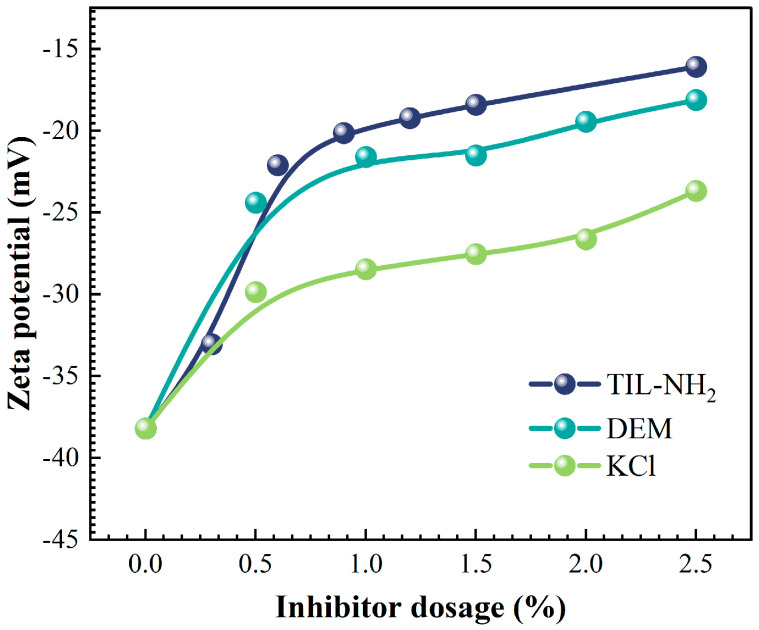
Effect of inhibitor type and concentration on the zeta potential of the shale pulverized slurry.

Figure 8 shows the effect of TIL-NH_2_, polyamine DEM, and KCl on the zeta potential of the clay mineral illite at different concentrations.

Figure 8 shows that the zeta potential of illite was −38.2 mV. As the inhibitor concentration increased, the absolute value of the zeta potential significantly decreased, indicating that the inhibitor reduced the surface charge of illite. TIL-NH_2_ significantly reduced the zeta potential of illite even at a lower concentration. At a 0.6% addition, the zeta potential was −22.1 mV, which was higher than the zeta potential values of polyamines DEM and KCl at a 1% addition. The absolute value of the zeta potential changed less at concentrations above 0.9%, remaining between −16 mV and −19 mV. This suggests that the ionic liquid significantly affects the diffusive bilayer state of illite agglomerates, reducing the tendency of water molecules to penetrate the illite layer.

The cationic structure on the TIL-NH_2_ polymer chain gives it polyelectrolyte properties with multiple charges, which are linearly adsorbed onto the negatively charged illite surface through charge interactions, electrostatic forces, and hydrogen bonding. The imidazole five-membered ring structure of TIL-NH_2_ eliminates the need for amine protonation and improves inhibition efficiency. The cationic groups in TIL-NH_2_ neutralize the negative charge on the surface of illite, reducing the electrostatic repulsive force between clay particles. The long-chain organic groups cover the hydrophilic groups on the surface of illite, reducing the structured water layer and lowering hydration swelling, thereby improving the mechanical properties of the shale.

#### 3.3.4. Infrared Spectral Analysis

**Figure 9:**
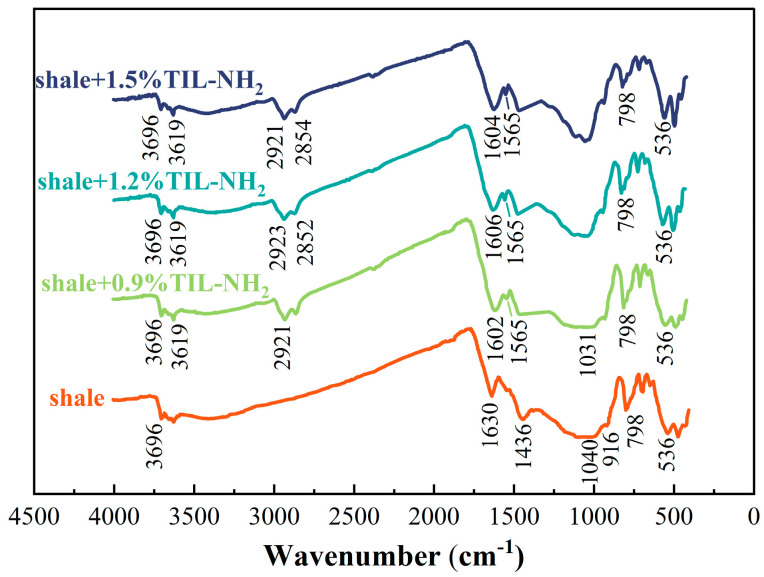
Infrared spectra of shale powder treated with different TIL-NH_2_ concentrations.

Figure 9 shows the infrared spectra of shale powder before and after treatment with different concentrations of TIL-NH_2_.

Figure 9 shows that the addition of TIL-NH_2_ did not change the main structure of the shale. The FT-IR spectra of the shale showed the stretching vibrational peak of O-H in the illite structure at 3695 cm^−1^, the characteristic O-H bending vibrational peak of water at 1634 cm^−1^, the bending vibrational peak of CH_3_ at 1435 cm^−1^, the stretching vibrational peak of Si-O at 1042 cm^−1^, and the bending vibrational peak of Al-OH at 918 cm^−1^. At TIL-NH_2_ concentrations of 0.6–1.5%, a new O-H stretching vibration absorption peak appeared at 3616 cm^−1^, and a shoulder signal of symmetric stretching vibration of the C-H bond in -CH_2_- was found at 2852 cm^−1^. Additionally, overlapping peaks of the N=C bond stretching vibration on the cationic imidazole ring, the N-H bond bending vibration in primary amines, and the N-H bending vibration in amides in the structure of TIL-NH_2_ were detected near 1565 cm^−1^.

These results indicate that the molecular chain of TIL-NH_2_ polymer adsorbs onto illite in shale. By comparing with the infrared spectra of TIL-NH_2_ molecules in Figure 2, it is observed that the intensity of the overlapping peaks around 1560 cm^−1^ is significantly weakened when TIL-NH_2_ fully interacts with shale powders, and a new peak appears around 1604 cm^−1^. This is because the N-H of the primary amide in TIL-NH_2_ forms a stabilized hydrogen-bonding structure with O on the surface of illite, causing a shift in the N-H vibrational frequency to higher frequencies. The original peak mainly consists of the N=C stretching vibration of the imidazole ring. The disappearance of the peak at 1630 cm^−1^ compared with the shale spectra suggests that TIL-NH_2_ reduces water adsorption on the surface of shale particles and between layers.

These results demonstrate the advantage of TIL-NH_2_ side group multifunctionality. The imidazole cation adsorbs on illite through electrostatic interaction, while the primary amine forms intermolecular interaction with the illite surface through hydrogen bonding. Together, they allow TIL-NH_2_ to adsorb tightly onto illite, preventing water molecules from entering and leading to the dispersion and hydration of illite. This reduces the repulsive force and hydration expansion force between shale powder particles, increasing their aggregation and stability, and suppressing shale dispersion. This explains the inherent reason for the aggregation of shale powder particles treated with TIL-NH_2_ in the particle size distribution experiments.

#### 3.3.5. XRD Analysis

**Figure 10:**
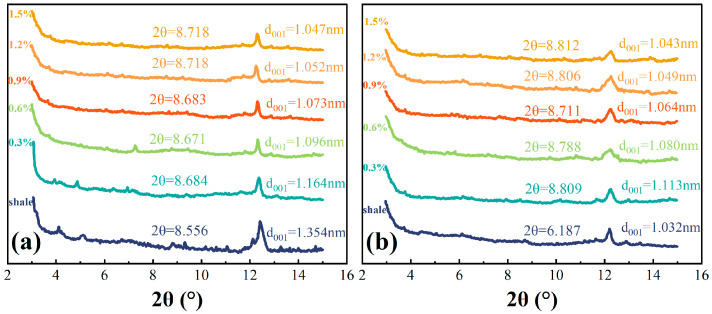
XRD spectra of shale powder treated with different concentrations of TIL-NH_2_ ((**a**) wet state, (**b**) dry state).

The X-ray diffraction spectral studies of the dry and wet shale powder samples determined in this section are shown in Figure 10.

Figure 10 shows that TIL-NH_2_ can effectively inhibit the hydration of illite, resulting in interlayer spacings of 1.032 nm and 1.354 nm for shale in dry and wet conditions, respectively. TIL-NH_2_ contains an imidazole cationic ring and a primary amine group, which adsorb strongly on the crystalline surfaces of illite to form dense and stable chemical bonds. This adsorption maintains charge equilibrium, prevents water from entering the interlayer, and reduces the interaction between water and the illite surface, leading to a reduction in interlayer spacing and the expulsion of interlayer water.

When the concentration of TIL-NH_2_ exceeds 0.9%, the interlayer spacing can be reduced to 1.073 nm. Unlike small molecule cations, the imidazole cationic ring is present in each repeating unit of TIL-NH_2_. When some cationic rings undergo electrostatic attraction between layers, other cationic rings are also induced to be attracted, achieving charge balance because of interactions and restrictions among chain segments. Additionally, the rigid and planar imidazole rings of TIL-NH_2_ can arrange in a tight monolayer structure among the layers to form a hydrophobic membrane.

At a higher concentration (1.5%), the interlayer spacings of shale in dry and wet states were 1.043 nm and 1.047 nm, respectively, and did not change significantly with increasing TIL-NH_2_ concentration. This indicates that the hydration inhibition effect of TIL-NH_2_ on illite reached saturation, reflecting the monolayer arrangement of TIL-NH_2_ in the interlayer voids.

#### 3.3.6. Thermogravimetric Analysis

**Figure 11:**
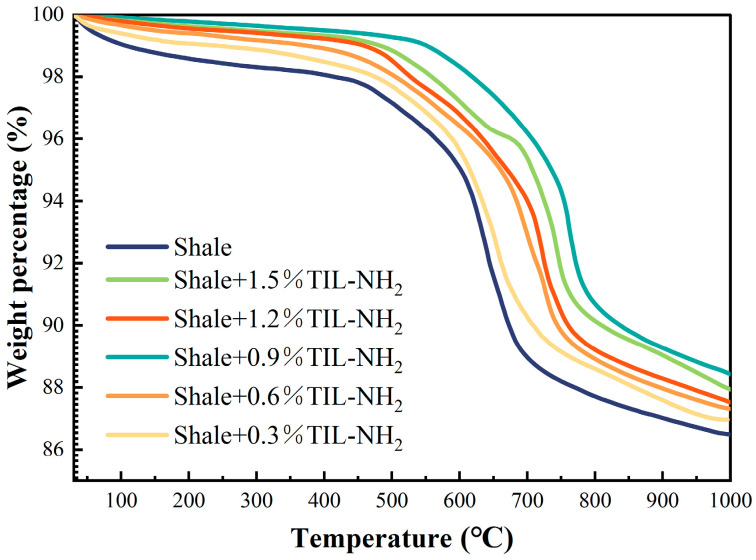
Heat weight loss curves of the shale powder after treatment with different concentrations of TIL-NH_2_.

In this section, thermogravimetric tests were carried out on pure shale flour and shale flour treated with 0.3–1.5% TIL-NH_2_, and the results are shown in Figure 11.

Figure 11 and Table 3 illustrate that TIL-NH_2_ significantly impacts the heat-loss curve and the content of various adsorbed waters in shale powders. According to the research by Xie Gang et al., different types of adsorbed water have specific weight loss temperature ranges as follows: free water loses weight between 70 °C and 80 °C, weakly bound water loses weight between 135 °C and 145 °C, and strongly bound water loses weight between 205 °C and 215 °C.

TIL-NH_2_, a multifunctional group inhibitor, exhibits a strong inhibition effect. At 80 °C, the free water content in the shale powder was calculated to be 0.7900%, the weakly bound water content was 0.4185%, and the strongly bound water content was 0.2646%. The low-concentration, high-efficiency property of TIL-NH_2_ is evident in lowering the weakly bound water content; when the TIL-NH_2_ concentration was 0.9%, the weakly bound water content dropped to 0.0951%, a reduction of over 78%. This aligns with previous analyses showing that TIL-NH_2_ adsorbs on the illite surface through cationic electrostatic interaction and primary amine hydrogen bonding, thereby compressing the diffusion layer and reducing the weakly bound water content.

Additionally, TIL-NH_2_ is temperature-resistant, and it reduces the strongly bound water content after treatment in the temperature range of 145 °C to 215 °C. The results were consistent across different TIL-NH_2_ concentrations, with each showing a reduction of more than 40% in strongly bound water. When the concentration of TIL-NH_2_ reached 0.9%, the shale powder had the most significant reduction in strongly bound water content, down to 0.0911%, which is more than 65% less than the blank shale powder. This indicates that TIL-NH_2_ replaces the water molecules that were originally strongly bound to the illite crystal surface, forming strong coordination with the illite lattice surface through electrostatic interactions and hydrogen bonding, thereby inhibiting the hydration degree on the illite surface. This conclusion is consistent with the reduced pattern of illite layer spacing shown by XRD tests.

#### 3.3.7. Micro-Morphological Analysis

**Figure 12:**
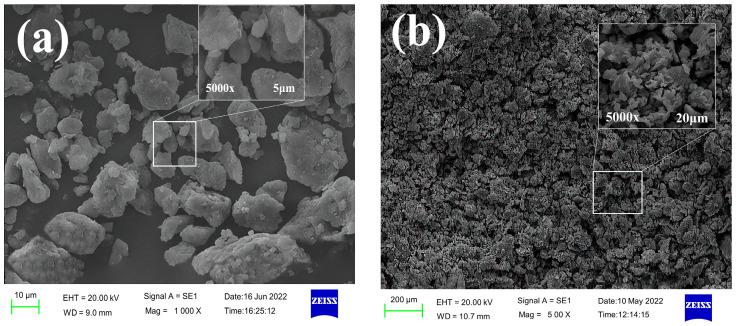

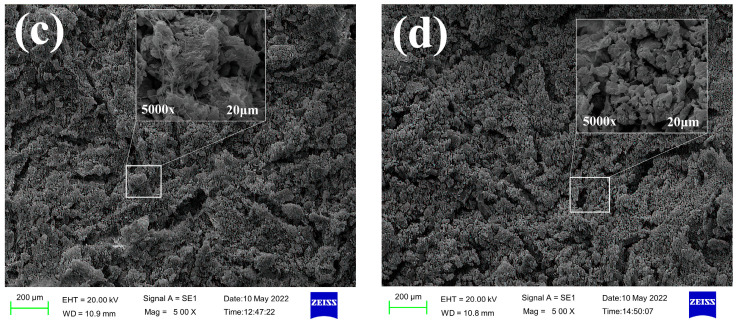
Wet state micromorphology of shale powder at different TIL-NH_2_ concentrations ((**a**) clear water, (**b**) 0.3%, (**c**) 0.9%, and (**d**) 1.5%).

In this section, environmental scanning electron microscopy (SEM) was used to analyze and study the microscopic interaction between the inhibitor and the shale powder. The resulting SEM images are shown in Figure 12.

Figure 12 shows that the wet shale powder in the clear water group was dispersed and had an uneven surface, while the wet shale powder treated with TIL-NH_2_ exhibited a special micro-morphology. After treatments with 0.3%, 0.9%, and 1.5% TIL-NH_2_, the size of the wet shale powder increased significantly, with magnification ranging from ×500 to ×5000. The inhibition effect approached saturation at a TIL-NH_2_ dosage of 0.9%, where the wet shale powder appeared as tightly stacked flower-like structures under ×500 magnification. These structures were not truly separated and remained connected despite voids.

The size of these clusters of wet shale powder exceeded 100 μm, consistent with the size distribution of the TIL-NH_2_ concentration. Under ×5000 magnification, the interaction among the wet shale powders was more clearly observed. Larger wet shale powders were covered with a thin film on the surface, indicating that the inhibitor molecules effectively encapsulated the wet shale powder particles. The TIL-NH_2_ molecules played an excellent encapsulation role. Additionally, larger wet shale powders attracted smaller ones to accumulate around them, connecting through linear stretching to form strong bonds. This reflects the aggregation and settling of shale powder particles observed in the particle size distribution experiments.

These results indicate that TIL-NH_2_ strongly adsorbs onto illite through bifunctional groups, greatly inhibiting the hydration and dispersion of wet shale powders and maintaining their tight structure. This shows that microscopic interactions are closely related to macroscopic changes.

### 3.4. Inhibition Mechanism of TIL-NH_2_

The design concept of TIL-NH_2_ is to impart versatility to the polymer from a molecular structure perspective. To achieve this, the monomer of TIL-NH_2_ needs diverse properties. The molecular structure is dendritic, with amine groups and imidazole cationic five-membered ring structures introduced on the side groups of the molecule. The amine group acts as an inhibitory functional group, while the imidazolium cationic five-membered ring structure possesses properties of both an inhibitory and a temperature-resistant functional group.

From the experimental analysis, it is evident that TIL-NH_2_ improves the wettability of water on shale powder and slows the rate of water penetration into illite. TIL-NH_2_ has a positive charge that counteracts the negative charge of illite, reducing the ionic concentration difference between the layers of illite. This brings the crystalline layers closer together, expelling the interlayer water, reducing the interlayer distance, and inhibiting the hydration of illite.

In the zeta potential test, TIL-NH_2_ adsorbed on the surface of illite and compressed the thickness of the diffusion layer around it. Infrared spectroscopy showed an increase in the frequency of N-H bond bending vibration peaks after the reaction of TIL-NH_2_ with shale powder, indicating that the amine group in TIL-NH_2_ formed a hydrogen bonding structure (N-H⋯O) with illite.

Thus, TIL-NH_2_ plays a dual inhibitory role: the imidazole cation adsorbs quickly and efficiently with illite, while the amine group at the end of TIL-NH_2_ forms a hydrogen bond with the oxygen atoms on the surface of illite. These effects are complementary and enhance the inhibition effect of TIL-NH_2_ on illite.

The reduction in various adsorbed water contents of shale powder by TIL-NH_2_ can be quantitatively measured by the heat loss weight test. The weakly bound water content is reduced from 0.4185% to 0.0951%, and the strongly bound water content is reduced from 0.2646% to 0.0911%. Scanning electron microscope images show that shale powder particles treated with TIL-NH_2_ exhibit an irregular bulk shape, with no obvious separation or cracks at lower magnifications, indicating low hydration and strong inter-particle bonding of illite.

**Figure 13:**
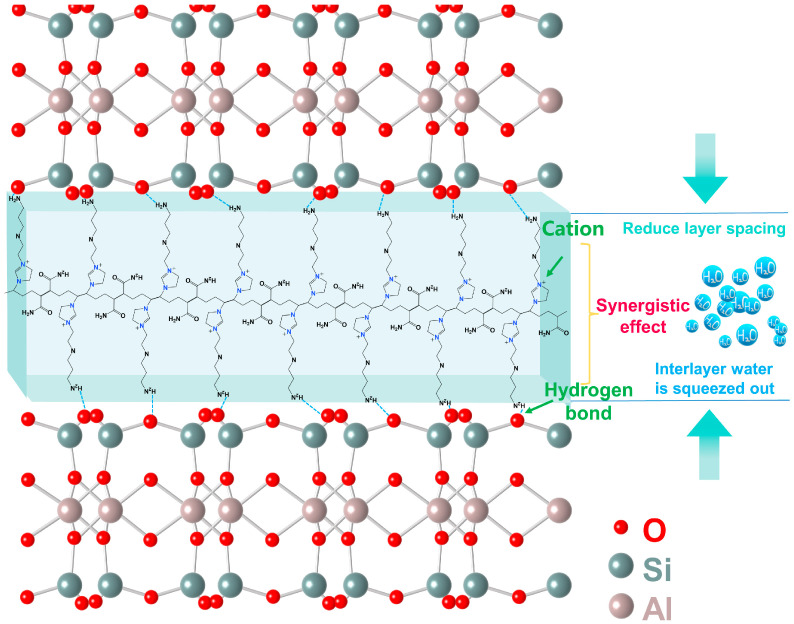
Schematic diagram of the inhibition mechanism of TIL-NH_2_ on the surface hydration of ilmenite.

Figure 13 provides a schematic diagram of the inhibition mechanism, explaining the mechanism of the inhibitory effect on illite.

In summary, the presence of primary amine groups and cationic imidazole rings is the fundamental reason for the inherent inhibition properties of the polymer inhibitor TIL-NH_2_. The adsorption mechanism between TIL-NH_2_ and illite involves both electrostatic interactions and hydrogen bonding, which work synergistically to enhance the inhibitory ability of TIL-NH_2_.

## 4. Conclusions

This study synthesized a new polyionic polymer inhibitor, TIL-NH_2_, and investigated its effects on the stability and hydration behavior of mud shale in shale gas horizontal wells. Various analytical techniques, including infrared spectroscopy, NMR spectroscopy, contact angle measurements, particle size distribution, zeta potential, X-ray diffraction, thermogravimetric analysis, and scanning electron microscopy, were used to analyze the structural characteristics and inhibition effects of TIL-NH_2_ comprehensively. The main conclusions are as follows:

(1) TIL-NH_2_ was synthesized via free radical polymerization of YX-NH_2_ and acrylamide, characterized by FTIR, NMR, and TGA. The resulting polymer exhibited an imidazole cationic structure and primary amine groups, providing excellent inhibition properties, temperature resistance, and chemical stability, with no significant thermal decomposition at 300 °C.

(2) TIL-NH_2_ significantly improved the wettability of shale, increasing the contact angle to 30.1°–41.2° at 0.3–1.5% concentration. At 0.9%, TIL-NH_2_ reduced the zeta potential from −38.2 mV to 16–19 mV and decreased the interlayer spacing from 1.354 nm to 1.047 nm. Infrared spectroscopy showed the formation of N-H⋯O hydrogen bonds, reducing water content. Thermal analysis revealed reductions in free, weakly bound, and strongly bound water, while SEM showed a compact structure, significantly inhibiting hydration swelling and dispersion.

(3) The inhibition mechanism of TIL-NH_2_ involves the following two key aspects: slowing water ingress into shale and forming strong electrostatic and hydrogen bonding interactions on the shale surface, resulting in efficient inhibition at low dosages.

## Figures and Tables

**Table 1:** Yield, purity, and inhibition effect of different batches of TIL-NH_2_.

Experiment Number	Yield (%)	Purity (HPLC) (%)	Inhibition Effect (Reduction in Shale Hydration Swelling) (%)
1	84.2	98.9	85.3
2	83.8	98.4	84.4
3	84.5	99.0	86.2

**Table 2:** Contact angle measurement results.

Soaking Time	Deionized Water Contact Angle (°)	Water-Based Drilling Fluid Contact Angle (°)	Oil-Based Drilling Fluid Contact Angle (°)	Synthetic-Based Drilling Fluid Contact Angle (°)
Initial	41.2	41.2	41.2	41.2
1 Day	40.8	40.5	40.9	40.7
7 Days	39.5	39.2	39.7	39.4
14 Days	37.0	36.8	37.2	36.9
30 Days	33.5	33.2	33.7	33.4

**Table 3:** Results of the adsorbed water content of shale powder after treatment with different concentrations of TIL-NH_2_.

Shale Powder Type	Type of Adsorbed Water		
	Free Water	Weakly Bound Water	Strong Bonded Water
Shale powder	0.7900%	0.4185%	0.2646%
0.3% TIL-NH_2_-treated shale powder	0.5255%	0.2742%	0.1587%
0.6% TIL-NH_2_-treated shale powder	0.2818%	0.2218%	0.1401%
0.9% TIL-NH_2_-treated shale powder	0.0538%	0.0951%	0.0911%
1.2% TIL-NH_2_-treated shale powder	0.1503%	0.1825%	0.1313%
1.5% TIL-NH_2_-treated shale powder	0.1333%	0.1443%	0.1273%

## Data Availability

The figures and tables used to support the findings of this study are included in this article.
